# Language in corticobasal syndrome: a systematic review

**DOI:** 10.1590/1980-57642021dn15-010002

**Published:** 2021

**Authors:** Isabel Junqueira de Almeida, Marcela Lima Silagi, Jacy Bezerra Parmera, Sonia Maria Dozzi Brucki, Eliane Schochat

**Affiliations:** 1 Department of Physical Therapy, Speech and Occupational Therapy, School of Medicine, Universidade de São Paulo – São Paulo SP, Brazil.; 2 Department of Human Communication Sciences, Universidade Federal de São Paulo – São Paulo, SP, Brazil.; 3 Department of Neurology, School of Medicine, Universidade de São Paulo – São Paulo, SP, Brazil.

**Keywords:** corticobasal syndrome, language, neurocognitive disorders, language tests, síndrome corticobasal, linguagem, transtornos neurocognitivos, testes de linguagem

## Abstract

Language is commonly impacted in corticobasal syndrome (CBS). However, the profile and type of language assessment in CBS are poorly studied.

**Objective:**

To identify language impairments in CBS.

**Methods:**

A search was performed in the Medline/PubMed database, according to the PRISMA criteria, using the keywords “corticobasal syndrome” OR “corticobasal degeneration” AND “language”. Articles on CBS covering language assessment that were written in English were included, with no constraints on the publication date.

**Results:**

A total of 259 articles were found and 35 were analyzed, consisting of 531 participants. Twenty-eight studies showed heterogeneous language deficits and seven mentioned nonfluent primary progressive aphasia. The most used tests were the Western Aphasia Battery (8 studies) and the Boston Naming Test (8 studies).

**Conclusion:**

It was not possible to identify a unique linguistic profile in CBS.

## RESUMO.

A linguagem encontra-se comumente alterada na síndrome corticobasal (SCB). No entanto, o perfil e a forma de avaliação da linguagem na SCB são pouco estudados.

**Objetivo:**

identificar as alterações de linguagem na SCB.

**Método:**

Realizou-se uma busca na base de dados Medline/PubMed, com as palavras-chave “síndrome corticobasal” OU “degeneração corticobasal” E “linguagem”. Artigos sobre SCB envolvendo avaliação de linguagem, escritos em inglês, foram incluídos, sem restrição de data de publicação.

**Resultados:**

Foram encontrados 259 artigos, e 35 estudos foram analisados, abrangendo 531 sujeitos. Um total de 28 estudos mostraram déficits heterogêneos de linguagem, e sete mencionaram afasia progressiva primária não-fluente. Os testes mais utilizados foram Western Aphasia Battery (8 estudos) e o Teste de Nomeação de Boston (8 estudos).

**Conclusão:**

Não foi possível identificar um perfil linguístico único em pacientes com SCB.

## INTRODUCTION

Corticobasal syndrome (CBS) is a progressive, neurodegenerative disease classified amongst atypical parkinsonian syndromes. The syndrome was first described in 1967 by Rebeiz, Kolodny, and Richardson, who presented three cases of patients with initial significant motor impairments followed by final stage cognitive impairments.^1^ The initial description focused on motor deficits and showed that cognitive impairments only occurred in the final stage, but it is now known that both can occur in equal proportion in CBS and may manifest as the first symptom.^2^ ^–^ ^6^

The terms “corticobasal syndrome” and “corticobasal degeneration” (CBD) represent distinct entities. The former denotes the clinical phenotype, whereas CBD is a pathological entity affecting cortical and subcortical regions, whose diagnosis can only be confirmed by *postmortem* anatomopathological analysis.^5^ An estimated 50% of patients with clinical symptoms of CBS are diagnosed with CBD at *postmortem*. In the remaining patients, tauopathies or amyloid pathology are generally found, such as Alzheimer's disease (AD). CBD is often found in patients clinically diagnosed with other syndromes.^5^ ^,^ ^7^ ^–^ ^9^

In CBS, classically, motor symptoms occur asymmetrically and include akinetic-rigid parkinsonism, dystonia, and myoclonic movements. Cognitive symptoms include apraxia, aphasia, cortical sensory deficits, and the alien hand phenomenon.^5^ ^,^ ^10^ ^,^ ^11^ This syndrome is generally challenging to diagnose owing to its clinical, pathological, radiological, and neuropsychological heterogeneity.^5^

Few studies have thoroughly investigated the profile of speech and language impairments in CBS. Some studies show a pattern similar to the nonfluent variant of primary progressive aphasia (nf-PPA), i.e., deficits at a morphosyntactic level, reduced fluency and apraxia of speech.^3^ ^,^ ^12^ ^–^ ^14^ However, other studies focusing on language assessment reveal a mixed pattern encompassing characteristics of more than one type of primary progressive aphasia (PPA).^15^ ^,^ ^16^

This heterogeneity found in the literature on speech and language in CBS may be explained by multiple factors: disease stage at the time of assessment, different underlying pathologies^6^ or lack of consensus on linguistic aspects to be assessed in these patients. Gorno-Tempini et al.^17^ recommended that language assessment in PPA cover the following domains: naming, word and sentence comprehension, word and sentence repetition, syntactic processing, semantic memory, reading, and motor aspects of speech.

The present review aimed to identify the language impairments in CBS patients.

## METHODS

The writing of this manuscript is in accordance with the Preferred Reporting Items for Systematic Reviews and Meta-Analysis (PRISMA) guidelines (www.prisma-statement.org), according to the following recommendations: introduction containing the description of the rationale and objectives of the review; methods containing the eligibility criteria, the information sources, the process for selecting studies, the data collection process, the definition of all variables for which data were sought, the methods used for assessing risk of bias of the studies, and how the results were analyzed; and discussion containing the summary of evidence, the limitations and the conclusions of the review.

The outcome of interest of this review is the profile of language in patients with CBS. Articles on CBS covering speech and language assessment were included, with no constraints on the publication date. Exclusion criteria were: 1) studies on CBD associated with syndromes other than CBS; 2) intervention studies in CBS; 3) studies written in languages other than Portuguese or English; 4) studies that could not be accessed via our University and were not open access.

The literature search was conducted using the electronic database Medline/PubMed, and it was based on manuscripts published up to February 2020. The keywords used were the following: “corticobasal syndrome” AND “language”, “corticobasal degeneration” AND “language”. The search was guided by the Population, Intervention, Comparison and Outcome (PICO) strategy. The population refers to the CBS patients, the intervention refers to the language assessment, the comparison is related to intragroup or between group comparisons, and the outcomes are the results from the language assessment.

All titles and abstracts were independently screened by two authors (IJA and MLS), according to the eligibility criteria previously established. The articles that were not excluded in this screening stage were fully read. A disagreement between the authors was resolved by consensus.

One author (IJA) extracted data from included studies and a second author (MLS) checked the information. Data were transferred to a data extraction sheet (using Microsoft Excel^®^) and included: 1) first author's name and year of publication; 2) sample size; 3) clinical and demographic data (gender, age, disease duration); 4) main speech and language results; and 5) speech and language tests used in the evaluation or speech and language abilities evaluated (when tests not mentioned). We classified the studies into three categories based on language evaluation:

- Comprehensive assessment: evaluation included all language domains recommended for testing PPA patients.^17^
- Restricted assessment: evaluation included some of the language domains recommended for testing PPA patients.^17^
- No tests or language skills mentioned: the tests or language skills evaluated were not reported.

Two authors (IJA and MLS) independently assessed the methodological quality and the risk of bias of the manuscripts included in this review through the JBI Critical Appraisal tool for cross-sectional studies.^18^ This tool has eight questions regarding the criteria for inclusion of the sample, the clarity of the description of the sample and the setting, the validity and reliability of the outcomes’ measurement, the appropriateness of statistical analysis and four questions that refer exclusively to clinical trial studies. Each question must be answered as “yes”, “no”, “unclear” or “not applicable”. All the questions regarding clinical trials were marked as “not applicable”. Each question that was marked as “yes” received 1 point. The question that refers to the outcome measurement was answered exclusively on the basis of the language evaluation described in each study. For most studies included, language was only one of the clinical characteristics assessed.

Discrepancies between the two authors were discussed until consensus was reached. All manuscripts were then classified into one of three groups, according to the score obtained on the JBI Critical Appraisal tool: “low quality”, if the study had less than 50% of the maximum score; “moderate quality”, for studies with 50 to 80% of the maximum score; and “high quality”, for studies with at least 80% of the maximum score.

Finally, confidence in the overall findings of the present review was assessed through the Confidence in the Evidence from Reviews of Qualitative Research (GRADE CERQual).^19^ This instrument is based on four components: 1) methodological limitations of the primary studies, 2) relevance of those studies to the review question, 3) coherence of results among primary studies, and 4) adequacy of data, i.e., the degree to which data support the review finding. From the analysis of these four components, the review may be classified as high confidence (“it is highly likely that the review finding is a reasonable representation of the phenomenon of interest”), moderate confidence (“it is likely that the review finding is a reasonable representation of the phenomenon of interest”), low confidence (“it is possible that the review finding is a reasonable representation of the phenomenon of interest”), and very low confidence (“it is not clear whether the review finding is a reasonable representation of the phenomenon of interest).^19^

The first component, methodological limitations, was judged using the JBI Critical Appraisal tool. Relevance, coherence and adequacy of data were judged exclusively on the basis of the language evaluations of primary studies.

Two authors (IJA and MLS) independently scored each component of the CERQual tool and its final classification. Discrepancies were discussed until consensus was reached.

## RESULTS

**Figure 1:**
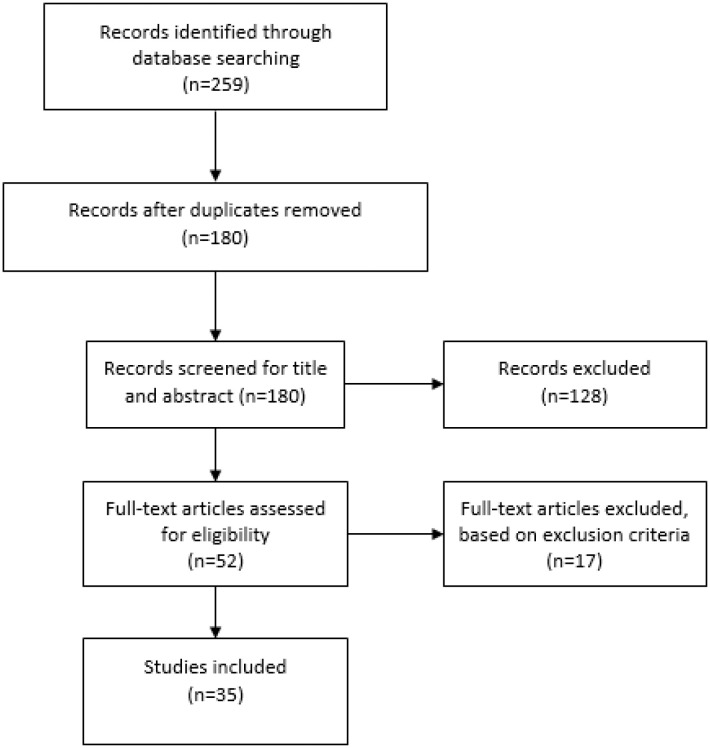
Literature search flow diagram.

The search on the Medline/PubMed database led to the retrieval of 259 articles, of which 79 were duplicate articles, giving a total of 180. After a screening of titles and abstracts, another 128 articles were excluded (literature reviews, letters to editor, articles in Japanese, studies on unrelated topics and inaccessible articles). A total of 52 articles were read in full, of which 17 were subsequently excluded (studies on CBD associated with syndromes other than CBS and studies on unrelated topics). We included 35 manuscripts in the present review (Figure 1).

Due to the heterogeneity of the population and outcomes of the studies included, it was not possible to perform a meta-analysis.

The demographic and clinical data of the studies are given in Table 1. The sample size was very heterogeneous, ranging from 1 to 55 CBS patients, with a median value of 11 and a mean of 15.2. CBS patient age ranged from 47 to 76 years, with a median of 66.2 and mean of 65.31 years. The mean number of female patients in the studies was slightly higher than that of male patients (12.14 and 8.9, respectively). Disease duration at the time of assessment ranged from 3 months to 8.08 years, with a median of 3.32 and mean of 3.46 years.

**Table 1:** Sociodemographic and clinical characteristics of studies selected.

Authors, year of publication	Sample size	Gender (male/female)	Age (years)	Disease duration (years)
Kertesz et al., 2000^2^	35	movement disorder=5/10 cognitive disorder=14/6	movement disorder=61.9 cognitive disorder=63.6	movement disorder=5.4 cognitive disorder=7.1
Frattali et al., 2000^42^	15	8/7	67.7	4.5
Graham et al., 2003^50^	10	7/3	67.6	3.35
Frattali et al., 2003^26^	prospective study=34 retrospective study=9	prospective study=18/16 retrospective study=4/5	prospective study=67.91 retrospective study=71.3	prospective study=3.8 retrospective study=2.78
Gorno-Tempini et al., 2004^20^	1	0/1	not applicable	not applicable
McMonagle et al., 2006^3^	55	motor onset=10/9 cognitive onset=16/20	n/a	motor onset=2.7 cognitive onset=3.6
McMillan et al., 2006^32^	16	n/a	66.3	n/a
Cotelli et al., 2006^29^	10	n/a	63.8	n/a
Cotelli et al., 2007^30^	11	n/a	64.6	n/a
Donovan et al., 2007^23^	1	0/1	60	4
Koenig et al., 2007^28^	experiment 1=8 experiment 2=9	experiment 1=3/5 experiment 2=5/4	experiment 1=64.5 experiment 2=70.1	n/a
Silveri and Ciccarelli, 2007^31^	5	2/3	63.8	1.6
Halpern et al., 2007^27^	16	9/7	67.07	3.9
Kim et al., 2008^45^	1	1/0	55	n/a
Shelley et al., 2009^12^	12	6/6	75.5	8.08
Gross et al., 2010^24^	20	9/11	67.4	3.9
Valverde et al., 2011^35^	1	0/1	74	0,25
Borroni et al., 2011^47^	30	21/9	63.5	2.5
Troiani et al., 2011^33^	11	n/a	65.5	n/a
Passov et al., 2011^36^	1	0/1	49	2
Dopper et al., 2011^41^	1	1/0	61	2
Caso et al., 2012^21^	2	0/2	case 1=64 case 2=70	case 1=2 case 2=4
Assal et al., 2012^37^	1	0/1	64	n/a
Mathew et al., 2012^4^	40	22/18	70	initial assessment=3 follow-up=4.9
Sakurai et al., 2013^38^	1	0/1	65	n/a
Burrell et al., 2013^15^	14	7/7	66.1	2.9
Turaga et al., 2013^51^	17	11/6	66.35	4.06
Marshall et al., 2015^40^	1	0/1	47	1
Abe et al., 2016^13^	26	9/17	76	2.3
Di Stefano et al., 2016^16^	45	23/22	69.2	3.2
Ash et al., 2016^34^	33	15/18	65.3	4.2
Kim et al., 2016^22^	1	0/1	58	4
Magdalinou et al., 2018^25^	4	n/a	n/a	n/a
Mazzon et al., 2018^39^	1	1/0	74	1
Dodich et al., 2019^14^	33	15/18	70.4	3.06

n/a: not available.

The profile of language impairments is given in Table 2. Seven studies (20%) cited nf-PPA as the predominant language deficit profile in patients with CBS.^4^ ^;^ ^12^ ^–^ ^14^ ^,^ ^20^ ^–^ ^22^ Twelve studies (34.28%) investigated specific aspects of language.^23^ ^–^ ^34^ In two (5.71%) studies, the language impairments were not described in detail.^26^ ^–^ ^35^ The remaining studies mentioned a variety of different symptoms, including agraphia^15^ ^,^ ^23^ ^,^ ^31^ ^,^ ^36^ ^–^ ^39^ speech apraxia,^2^ ^,^ ^23^ ^,^ ^36^ ^,^ ^37^ ^,^ ^39^ ^,^ ^40^ dysarthria,^36^ ^,^ ^41^ a mixed type of PPA,^16^ logopenic variant of PPA (L-PPA),^15^ ^,^ ^21^ anomic aphasia,^3^ ^,^ ^4^ ^,^ ^42^ transcortical motor aphasia^42^ and Broca's aphasia.^42^

**Table 2:** Profile of speech-language impairments, tests used for assessment, type of evaluation employed, and quality of studies included.

Authors, year of publication	Main speech/language results	Speech and language tests or abilities tested	Classification of the language evaluation	Quality of studies
Kertesz et al., 2000^2^	Initially, only word finding difficulties; verbal apraxia in 3/35 patients	WAB	Comprehensive assessment	High
Frattali et al., 2000^42^	Anomic, Broca's and transcortical motor aphasia	WAB (1st section)	Restricted assessment	Moderate
Graham et al., 2003^50^	Specific linguistic deficit involving phonologic processing	Letter fluency (FAS), semantic fluency, picture naming, word-picture matching, PPT, Single-word reading (The surface list), nonword reading, oral spelling, phoneme blending and phoneme segmentation	Restricted assessment	Moderate
Frattali et al., 2003^26^	Aphasia, without details	WAB (1st section)	Restricted assessment	Moderate
Gorno-Tempini et al., 2004^20^	nf-PPA	Motor speech evaluation, BDAE (verbal agility component, repetition), WAB (spontaneous speech section, written picture description, repetition, auditory word recognition, sequential command), BNT, PPT, CYCLE-R, PALPA (Regularity and Reading, Lexical Morphology and Grammatical Class, Homophone Decision), Gathercole and Baddeley's Non-Word Repetition task	Comprehensive assessment	High
McMonagle; Blair; Kertesz, 2006^3^	Majority classification of anomic aphasia (55%) in both groups (cognitive and motor onset), but more motor onset patients were normal and more cognitive onset patients had severe aphasias	WAB (1st section)	Restricted assessment	Moderate
McMillan et al., 2006^32^	Non-aphasic patients with CBD are significantly impaired in their comprehension of quantifiers	Sentence comprehension task	Restricted assessment	Moderate
Cotelli et al., 2006^29^	Action naming is impaired in FTD, PSP and CBS in comparison to object naming	Token Test, phonemic and semantic verbal fluency, action and object naming, Battery for Analysis of the Aphasic Deficits (action–object comprehension tasks)	Restricted assessment	Moderate
Cotelli et al., 2007^30^	CBS patients present with syntactic knowledge deficits	AAT (repetition, naming, writing and comprehension), BADA (sentence comprehension tasks)	Comprehensive assessment	Moderate
Donovan et al., 2007^23^	Aphasia, speech apraxia, alexia, agraphia, social language usage deficits	Pragmatic Protocol, Revised Token Test, WAB, BNT, Battery of Adult Reading Function, Woodcock Reading Mastery Tests, Comprehensive Test of Phonological Processing	Comprehensive assessment	High
Koenig et al., 2007^28^	CBS patients were impaired in similarity-based categorization process	Semantic decision task	Restricted assessment	Moderate
Silveri and Ciccarelli, 2007^31^	Hypofluent speech, agrammatism, anomia, word-finding difficulties, agraphia	Confrontation naming task of objects and verbs, semantic and phonemic fluency	Restricted assessment	Moderate
Halpern et al., 2007^27^	CBS patients were less accurate and slower at judging smaller Arabic numeral dot array compared to FTD patients and controls	PPT	Restricted assessment	Moderate
Kim et al., 2008^45^	Language functions relatively preserved	BNT	Restricted assessment	Low
Shelley et al., 2009^12^	nf-PPA	n/a	No tests or language skills are mentioned	Low
Gross et al., 2010^24^	CBS patients have a higher-level deficit integrating described events into a coherent narrative	BNT, PPT	Restricted assessment	Moderate
Valverde et al., 2011^35^	Aphasic, without details	n/a	No tests or language skills are mentioned	Moderate
Borroni et al., 2011^47^	The AD-like group showed greater impairment of memory performances, language and psychomotor speed while the nAD-like group had more severe extrapyramidal syndrome	Semantic and phonemic verbal fluency, Token Test,	Restricted assessment	Moderate
Troiani et al., 2011^33^	CBS patients were significantly impaired in their judgments of quantified statements	Philadelphia Brief Assessment of Cognition (used to exclude aphasic patients), BNT, phonemic verbal fluency (FAS), Oral Sentence Comprehension Test, short sentence comprehension task	Restricted assessment	Moderate
Passov et al., 2011^36^	Mild apraxia of speech, mild hypokinetic dysarthria, apraxic agraphia	“Formal speech pathology evaluation”; picture description task; confrontation naming task; comprehension of simple and complex commands; writing; spelling; motor speech disorders	Comprehensive assessment	High
Dopper et al., 2011^41^	nonfluent speech with perseverations, word-finding difficulties and comprehension deficits, hypokinetic dysarthria	n/a	No tests or language skills are mentioned	Moderate
Caso et al., 2012^21^	nf-PPA, L-PPA	AAT, Token Test, phonemic and semantic verbal fluency	Comprehensive assessment	High
Assal et al., 2012^37^	crossed-PAOS followed by peripheral agraphia	Bachy 90-item battery (confrontation naming), MTL (auditory and written language comprehension, and writing), written descriptions of the Bank Robbery Picture, and the Cookie Theft Picture, and oral spelling with the French version of the WAIS III	Comprehensive assessment	Moderate
Mathew et al., 2012^4^	nf-PPA (60%) and anomic aphasia (40%)	n/a	No tests or language skills are mentioned	Moderate
Sakurai et al., 2013^38^	Progressive apraxic agraphia with micrographia, and acalculia	WAB, reading and writing test with 100 single-character kanji and kana transcription	Comprehensive assessment	Moderate
Burrell et al., 2013^15^	Impaired single word repetition (61.5%), dysgraphia (58.3%), phonological errors in spontaneous speech (46.2%), impaired sentence repetition (38.5%), and word-finding difficulty (30.8%). Agrammatism and anomia were only occasionally identified. There was a trend for greater impairment of sentence repetition in PiB-positive cases	Motor speech disorder, phonological errors, agrammatism, word-finding difficulty, anomia, word and sentence repetition	Restricted assessment	Moderate
Turaga et al., 2013^51^	phonemic verbal fluency impairment	ACE-R (phonemic verbal fluency, semantic verbal fluency, naming)	Restricted assessment	Moderate
Marshall et al., 2015^40^	PAOS	n/a	No tests or language skills are mentioned	Moderate
Abe et al., 2016^13^	nf-PPA (34,61%)	Standard Language Test of Aphasia	Comprehensive assessment	High
Di Stefano et al., 2016^16^	Mixed progressive aphasia, including disorders of L-PPA (anomia, sentence repetition impairment) and S-PPA (deficits in single-word comprehension)	BDAE, picture naming test, single-word comprehension task, semantic and phonemic verbal fluency, sentence repetition test, assessment of motor speech disorders and agrammatism	Comprehensive assessment	High
Ash et al., 2016^34^	CBS were significantly impaired in the production of quantifiers	BNT, semantic verbal fluency, semi-structured speech sample (description of the Cookie Theft picture from the BDAE)	Restricted assessment	Moderate
Kim et al., 2016^22^	nf-PPA	WAB, BNT, semantic and phonemic verbal fluency	Comprehensive assessment	High
Magdalinou et al., 2018^25^	Impaired verbal fluency and sentence generation	BNT, Graded Naming Test, Verb Naming Task, PALPA (sentence comprehension), Sentence Production Program for Aphasia (expressive grammar), phonemic and semantic verbal fluency, National Adult Reading Test, sentence completion tasks	Restricted assessment	Moderate
Mazzon et al., 2018^39^	Apraxia of speech, characterized by slow overall speech rate, mild dysphonia, abnormal prosody, distorted and inconsistent speech sound substitutions, segmentation of syllables in words productions, mild dysgraphia with letter substitutions and omissions	Motor Speech Evaluation, AAT, Cookie Thief Test	Comprehensive assessment	High
Dodich et al., 2019^14^	nf-PPA, other language disorders	Connected speech production (speech apraxia and articulation difficulties, anomia, circumlocutions, agrammatism), CAGI battery (naming and word-picture matching), phonemic and semantic controlled associations, AAT (repetition), Token Test, BADA (sentence comprehension) phonemic (P-F-L) and semantic (animals-fruits-cars) verbal fluency	Comprehensive assessment	High

AAT: Aachen Aphasia Test; ACE-R: Addenbrooke's Cognitive Examination – revised; AD: Alzheimer's disease; BADA: Batteria per l’Analisi dei Deficit Afasici; BDAE: Boston Diagnostic Aphasia Examination; BNT: Boston Naming Test; CBD: corticobasal degeneration; CBS: Corticobasal syndrome; CYCLE-R: Curtiss-Yamada Comprehensive Language Evaluation-Receptive; FTD: frontotemporal degeneration; L-PPA: logopenic variant of primary progressive aphasia; MTL: Montreal-Toulouse Language Assessment Battery; n/a: not available; nAD: non-Alzheimer's disease; nf-PPA: Nonfluent variant of primary progressive aphasia; PALPA: Psycholinguistic Assessments of Language Processing in Aphasia; PAOS: Progressive apraxia of speech; PP: Pragmatic Protocol; PPA: primary progressive aphasia; PPT: Pyramids and Palm Trees; WAB: Western Aphasia Battery.

The tests used for assessment and classification of type of evaluation are also given in Table 2. The most frequently used tests in the studies were the Western Aphasia Battery (WAB)^2^ ^,^ ^3^ ^,^ ^20^ ^,^ ^22^ ^,^ ^23^ ^,^ ^26^ ^,^ ^38^ ^,^ ^42^ ^,^ ^43^ and the Boston Naming Test (BNT),^20^ ^,^ ^22^ ^–^ ^25^ ^,^ ^33^ ^,^ ^34^ ^,^ ^44^ ^,^ ^45^ both mentioned by eight studies (22.85%). The Token Test^46^ was used in five studies (14.28%)^14^ ^,^ ^21^ ^,^ ^23^ ^,^ ^29^ ^,^ ^47^ and the Aachen Aphasia Test (AAT^48^)^14^ ^,^ ^21^ ^,^ ^30^ ^,^ ^39^ and Pyramids and Palm Trees (PPT)^20^ ^,^ ^24^ ^,^ ^27^ ^,^ ^49^ ^,^ ^50^ featured in four articles (11.42%).

Regarding the type of evaluation employed in the studies, 13 (37.14%) used a comprehension speech/language assessment,^2^ ^,^ ^13^ ^,^ ^14^ ^,^ ^16^ ^,^ ^20^ ^–^ ^23^ ^,^ ^30^ ^,^ ^36^ ^–^ ^39^ 17 (48.57%) used a restricted assessment,^3^ ^,^ ^15^ ^,^ ^24^ ^,^ ^29^ ^,^ ^31^ ^–^ ^34^ ^,^ ^42^ ^,^ ^45^ ^,^ ^47^ ^,^ ^50^ ^,^ ^51^ while five (14.28%) failed to mention the tests or language skills evaluated.^4^ ^,^ ^12^ ^,^ ^35^ ^,^ ^40^ ^,^ ^41^

The assessment of methodological quality of the manuscripts is shown in Table 2. Ten studies (28.57%) were classified as “high quality”,^2^ ^,^ ^13^ ^,^ ^14^ ^,^ ^16^ ^,^ ^20^ ^–^ ^23^ ^,^ ^36^ ^,^ ^39^ 23 (65.71%) as “moderate quality”,^3^ ^,^ ^4^ ^,^ ^15^ ^,^ ^24^ ^–^ ^35^ ^,^ ^37^ ^,^ ^38^ ^,^ ^40^ ^–^ ^42^ ^,^ ^47^ ^,^ ^50^ ^,^ ^51^ and two (5.71%) as “low quality”.^12^ ^,^ ^45^

GRADE CERQual analysis was carried out for three separate review findings: comprehensive language impairments, impairment in isolated language processing, and absence of language impairment. The overall CERQual assessment of confidence in the results was considered low for the first two review findings and very low for the last one (Table 3).

**Table 3:** Confidence in the Evidence from Reviews of Qualitative Research assessment of review findings.

Review findings	Studies contributing to the review finding	Methodological limitation	Relevance	Coherence	Adequacy of data	Overall CERQual assessment of confidence
Comprehensive language impairments (presence of aphasia)	2; 3; 4; 12; 13; 14; 15; 16; 20; 21; 22; 23; 35; 41; 42; 47; 50	minor methodological limitation (8 studies with moderate methodological quality and 1 study with low methodological quality)	moderate concerns about relevance (only 8 studies carried out a comprehensive language assessment)	moderate concerns about coherence (inconsistent data across studies regarding language outcomes)	substantial concerns about adequacy of data (6 studies are case reports or case series and 4 have up to 15 participants)	Low confidence
Impairments in isolated language processing	24; 25; 26; 27; 28; 29; 30; 31; 32; 33; 34; 36; 37; 38; 39; 40; 51	moderate methodological limitation (15 studies with moderate methodological quality)	moderate concerns about relevance (only 5 studies carried out a comprehensive language assessment)	moderate concerns about coherence (inconsistent data across studies regarding language outcomes)	substantial concerns about adequacy of data (7 studies are case reports or case series)	Low confidence
Absence of language impairments	45	substantial concerns (low methodological quality)	substantial concerns about relevance (restricted language assessment)	not applicable	substantial concerns about adequacy of data (case report)	Very low confidence

CERQual: Confidence in the Evidence from Reviews of Qualitative Research.

## DISCUSSION

The purpose of the present literature review was to identify a possible language impairment profile in patients with CBS.

First, regarding the demographic and clinical characteristics of the sample, the mean age of patients was 65.31 years. The slight predominance of more women in studies is in line with the literature,^7^ though some studies found no evidence of gender differences.^9^ ^,^ ^52^ ^,^ ^53^ The sample size was relatively small, with a median value of 11 subjects. This may be explained by the rarity of the syndrome.

Regarding the language profile in CBS, many studies cited the nf-PPA phenotype as a common feature. This profile was found in 20% of the articles.^4^ ^,^ ^12^ ^–^ ^14^ ^,^ ^20^ ^–^ ^22^ Although not a high rate, this phenotype appears to be the most common. Other studies cited a broad range of profiles, which are discussed below.

Frattali and colleagues^42^ sought to characterize language profiles in 15 CBS patients. They were classified as having anomic aphasia, Broca's aphasia, or transcortical motor aphasia.

Another study with a similar objective, conducted by Graham,^50^ detected language deficits mainly in phonological awareness, spelling and verbal fluency tests, suggesting language impairments related to phonological processing.

Three studies that explored the relationship between clinical aspects and the underlying pathology found different language profiles. Borroni and colleagues^47^ assessed 30 patients with CBS, divided into two groups according to results on cerebral spinal fluid (CSF) examination (suggestive of AD and not suggestive of AD). The probable AD group showed more significant impairment on tests of episodic memory and language comprehension, whereas the other group showed more severe extrapyramidal abnormalities. However, language assessment was restricted to a sentence comprehension test (Token Test) and verbal fluency tests.

Burrell and colleagues^15^ assessed 14 CBS patients, divided into two groups according to the probable underlying pathology based on amyloid positron emission tomography (PET). The authors found language impairments in the following decreasing order of frequency: word repetition, dysgraphia, sound substitution in spontaneous speech, sentence repetition, and word-finding difficulties. The group with probable AD had a more marked problem on sentence repetition, a characteristic of L-PPA, whose underlying pathology is typically AD. The authors correlated difficulty in sentence repetition with a higher likelihood of AD being the underlying pathology.

In the study by Di Stefano and colleagues,^16^ 45 CBS patients were assessed with a comprehensive language battery. Language impairment was the most prevalent cognitive deficit in the sample. Language deficits were found in the following tasks: phonemic and semantic verbal fluency, sentence repetition, and word comprehension. Patients with CSF biomarkers indicating probable AD as underlying pathology showed a positive correlation with Gerstmann syndrome, and the group without AD presented more severe language deficits, especially in picture naming and word comprehension. The authors suggested a mixed aphasia phenotype, including characteristics of L-PPA and the semantic variant of PPA (S-PPA).

The language heterogeneity in CBS was also illustrated in some case reports. Sakurai et al.^38^ reported the case of a patient with CBS and apraxic agraphia and micrographia, without other language impairments, detected using a comprehensive language assessment.

Mazzon and colleagues^39^ reported the case of a 74-year-old man, who evolved with language impairments, compatible with nf-PPA and apraxic agraphia.

Another case of apraxic agraphia was reported by Passov and colleagues.^36^ In this case, apraxic agraphia was the onset symptom. The patient evolved with motor and speech disturbances (hypokinetic dysarthria and speech apraxia).

Assal and colleagues^37^ reported the case of a patient with progressive apraxia of speech who evolved with peripheral agraphia and, subsequently, with characteristic CBS symptoms. Imaging scans disclosed hypometabolism and atrophy in the right hemisphere, confirming a case of crossed-apraxia of speech.

In summary, although the nf-PPA phenotype seems to be the most common language profile in CBS, it is possible to find characteristics of L-PPA as well as S-PPA. Other language characteristics, such as writing impairments, difficulty in comprehension and expression of quantifiers (words preceding nouns that convey quantity information), syntactic processing impairment, and deficits in narrative skills may also be present in CBS patients. A review of language in CBS also reported a wide array of language profiles.^6^

Regarding the tests used in the assessment of language impairments in CBS, WAB was the most utilized comprehensive language test in the studies reviewed.^2^ ^,^ ^3^ ^,^ ^20^ ^,^ ^22^ ^–^ ^3^ ^,^ ^26^ ^,^ ^38^ ^,^ ^42^ WAB assesses the following linguistic abilities: speech content, fluency, auditory comprehension, repetition, naming, reading, and writing. It also includes the assessment of non-linguistic skills in its second part: apraxia, calculation, and constructional and visuospatial abilities. Three composite scores can be obtained from WAB: Aphasia Quotient (AQ), Language Quotient (LQ), and Cortical Quotient (CQ). AQ is derived from spontaneous speech, auditory verbal comprehension, repetition, and naming and word-finding tests. It is a widely used measure of aphasia severity. LQ includes, in addition to the abilities covered in AQ, reading and writing, and CQ is derived from the whole test.

A study^54^ investigated the use of the revised version of WAB (WAB-R)^55^ for detecting PPA subtypes. A total of 169 patients were included, with different PPA subtypes and progressive apraxia of speech (PAOS). On group comparisons, the AQ proved satisfactory for distinguishing PPA subtypes from PAOS. At the individual level, however, sensitivity for detecting aphasia proved low, as 20% of the PPA participants had AQ in the normal range. The authors concluded that, for PPA, WAB-R should be used together with other tests, including an assessment for motor speech disorders.

Another widely used test for language evaluation on CBS was the BNT, mentioned in eight studies.^20^ ^,^ ^22^ ^–^ ^25^ ^,^ ^33^ ^,^ ^34^ ^,^ ^45^ BNT is a visual confrontation naming test that assesses lexical access and the semantic system.

In one^45^ of the eight studies that used BNT, this test was used alone to evaluate language abilities. In other studies, BNT was used as part of a larger battery of language tests.

The Token Test, which was utilized in five studies,^14^ ^,^ ^21^ ^,^ ^23^ ^,^ ^29^ ^,^ ^47^ also assesses a specific language ability, i.e., verbal comprehension, including simple and complex sentences. Again, except for one study,^47^ the others used the Token Test as part of a larger language battery.^14^ ^,^ ^21^ ^,^ ^23^ ^,^ ^29^

AAT, like WAB, is a comprehensive language assessment battery, initially developed in German. AAT includes the assessment of spontaneous language, verbal comprehension, repetition (words and phrases of increasing length), reading and writing, and naming abilities. The four studies that included this test were conducted in Italian universities, and used the Italian version.^14^ ^,^ ^21^ ^,^ ^30^ ^,^ ^39^

PPT is a semantic access test. It consists of pictures of objects presented in triads, in which the one on the top must be matched to one of two others (the distractor or the target picture), on the basis of some type of association, which varies across the triads. The distractor and the target pictures are always semantic coordinates. PPT comprises 52 triads. This test has the advantage of not requiring a verbal response, which is very useful to assess semantic knowledge in patients with severe aphasia or motor speech disorders.

PPT was part of a larger language battery in three of the four studies that utilized it.^20^ ^,^ ^24^ ^,^ ^50^ In the survey conducted by Halpern et al.,^27^ the language assessment, however, was based exclusively on the PPT score. Nevertheless, this study aimed to assess the semantic knowledge of numbers.

Regarding the type of evaluation used in the assessment of language impairments in CBS, results showed that just over a third of the studies included in this review performed a comprehensive assessment,^2^ ^,^ ^13^ ^,^ ^14^ ^,^ ^16^ ^,^ ^20^ ^–^ ^23^ ^,^ ^30^ ^,^ ^36^ ^–^ ^39^ in strict compliance with recommended guidelines for assessing PPA.^17^

Of the studies performing a restricted assessment, some sought to analyze specific aspects of language. Frattali and colleagues^26^ investigated the occurrence of yes/no reversal phenomenon in CBS; in other words, when a patient verbalizes or gestures “no” when meaning “yes”, or vice versa. This error was found in almost half of the sample and was attributed to deficits in inhibitory control and mental flexibility.

Three studies by the same group^32^ ^–^ ^34^ investigated comprehension and expression of quantifiers, showing that CBS patients had significantly worse performance in comprehension and expression of quantifiers compared to controls. In all of those three studies, patients were non-aphasic as inclusion criteria, and they were tested on only a few linguistic abilities.

Three other studies focused on verb and syntactic processing in CBS.^29^ ^–^ ^31^ CBS patients had more significant impairment in processing verbs than nouns and in syntactic knowledge.

One study^28^ investigated semantic memory processing in AD patients, comparing them with CBS patients. The task consisted of similarity-based and rule-based processes for teaching names of non-existent, but biologically plausible animals. CBS patients were impaired in both learning strategies, with disadvantages in the similarity-based processing, as they tended to focus on a single element of the picture.

The narrative skills of CBS patients were investigated by Gross et al.,^24^ using a story-telling task based on a book of images. CBS patients displayed impaired discursive abilities, with deficits in organization and coherence, having difficulties integrating elements described into a coherent narrative. The formal aspects of language were not specified in the study.

Another study^25^ was based on the notion that patients with CBS, PSP and Parkinson's disease (PD) have reduced verbal output and decreased ability to produce new information, in the absence of other language deficits, a condition referred to as “dynamic aphasia”. The authors used tasks that involved generating new information in different situations with an increasing level of difficulty. All patients were impaired in producing sentences from a context and describing pictures.

Halpern et al.^27^ compared the number knowledge of CBS patients with those with frontotemporal degeneration (FTD). Patients had to state whether a given Arabic numeral matched the number of black circles displayed on a screen. The stimuli were divided into “low numbers” (2–4) and “high numbers” (5–9). Patients with CBS had worse performance compared to the FTD group, particularly for low numbers, showing impairment in semantic knowledge of numeric values. The patients were described as non-aphasics.

Finally, this diversity of linguistic profiles in CBS is partly due to its clinical-pathological heterogeneity.^6^ Some recent articles aimed to identify clinical characteristics indicative of the underlying pathology of CBS, including language characteristics. These articles may call attention to the importance of a comprehensive language assessment, since, in some of these studies, correlations were found between specific language deficits and the biomarker for AD, showing that the linguistic profile may be useful in the identification of the underlying pathology.

However, this review shows that there are still few studies that comprise a complete assessment of language. Moreover, part of the studies included in this review were case reports or studies with a small sample. A higher number of studies with comprehensive language assessment are necessary to clarify the language profile of CBS patients.

The assessment of the methodological quality of the studies showed that less than a third were classified as “high quality”.^2^ ^,^ ^13^ ^–^ ^14^ ^,^ ^16^ ^,^ ^20^ ^–^ ^23^ ^,^ ^36^ ^,^ ^39^ Among the studies classified as “moderate quality”,^3^ ^,^ ^4^ ^,^ ^15^ ^,^ ^24^ ^–^ ^35^ ^,^ ^37^ ^,^ ^38^ ^,^ ^40^ ^–^ ^42^ ^,^ ^47^ ^,^ ^50^ ^,^ ^51^ the majority lost points on the item regarding outcome evaluation, which, here, refers to the language evaluation. This is in line with the classification of the type of evaluation discussed above.

The overall CERQual assessment of confidence in the outcomes of this review was considered low for the findings concerning comprehensive language impairments (presence of aphasia) and impairments in isolated language processing, and very low for the findings concerning absence of language impairments. This is mainly due to the adequacy of data. Fourteen studies were case reports or case series, and some included less than 15 patients. There were also concerns about relevance, as few studies carried out a comprehensive language assessment, and coherence, as the results regarding language were inconsistent across studies. Some studies had methodological limitations.

The main limitation of this review refers to the search, which was performed in only one database. A more exhaustive search would possibly result in more studies with comprehensive language assessment, that could help in delineating the language profile of CBS patients. One possible future direction for a primary study is a more detailed analysis of the motor speech disorders and their form of assessment in CBS. It is well documented that patients with CBS may present with dysarthria and/or apraxia of speech.

The results of the present review showed that the language impairments found in patients with CBS were heterogeneous. Concerning the language assessment, the most used tests for evaluation were WAB and BNT. Finally, most publications were based on restricted language assessments and had moderate methodological quality. Therefore, the data available in the relevant literature are insufficient to identify a single language profile in CBS patients.
